# The Treatment of Appendicitis

**Published:** 1903-12-12

**Authors:** 


### THE TREATMENT OF APPENDICITIS.

Speaking of the importance and difficulty of early diagnosis and prognosis in appendicitis in a lecture at the West London Hospital, Mr. Stephen Paget [1] said that during the first 12 hours it was possible, in most cases, to prognosticate whether a patient with pain in his right iliac fossa had a colic or appendicitis, and whether the form of inflammation was catarrhal or ulcerative. Some cases cannot wait 12 hours; in these the signs of perforation of the appendix are evident. The patient is faint, restless, and cold, vomiting is continuous, and the pulse rapid and weak; also there is great tenderness in the right iliac region. In less severe cases the pain, at first general, becomes localised to the right iliac and umbilical regions. Vomiting is occasional. If the pulse-rate 12 hours after the onset is 100 when it should be 80, if there is great tenderness in McBurney's point region, and if the patient, though feeling fairly well, looks ill and is drowsy, operation should not be delayed. Distended coils of intestine lying inert and mapped out behind the abdominal wall and almost immobile abdominal muscles when noticed should urge the operator not to delay. If at the end of 12 hours, with rest and warmth in bed and hot fomentations but without opiate, the patient is in no way improved, the risk of operation becomes less formidable than the risk of delay. The two reasons for waiting are: (1) The attack may be catarrhal and subside, (2) it may be suppurative, and waiting allows adhesion time to form. These two views of the case exclude one another; therefore, they must be considered separately. If the onset is sudden, and the patient is rather better after 12 hours, do not operate, but watch for the quickened pulse and point of extreme tenderness. It is true that patients do recover after operation, though they have been kept for days with a gangrenous appendix inside them soaked in fœtid pus; but many lives are lost by waiting, and the balance of disastrous cases of septic peritonitis is not on the side of those who operate early. Many cases come to the surgeon when an appendix abscess has formed on the third or fourth day of disease. In these the best treatment is to open the abscess by an incision over the outer side of the abdominal swelling and keeping as far as possible to the outer side of the lump. When opening the peritoneum a sufficiently large wound should be made. In case of a thick-walled abscess with much surrounding adhesions the cavity should be gently washed out and the appendix left. If there is no continuous wall and adhesions are incomplete the appendix should be removed, the cavity mopped out with gauze and packed with moist strips, one to the stump of the appendix, one down to the brim of the pelvis, and one towards the middle line.

[1] West London Medical Journal, October, 1903.

